# The influence of meteorological factors on tuberculosis incidence in Southwest China from 2006 to 2015

**DOI:** 10.1038/s41598-018-28426-6

**Published:** 2018-07-03

**Authors:** Yuanyuan Xiao, Limei He, Ying Chen, Qinying Wang, Qiong Meng, Wei Chang, Lifen Xiong, Zhen Yu

**Affiliations:** 1 Department of Epidemiology and Health Statistics, School of Public Health, Kunming Medical University, Kunming, Yunnan China; 2 Division of Chronic Infectious Disease Prevention and Control, Jinghong Municipal Center for Disease Control and Prevention, Jinghong, Xishuangbanna Dai Autonomous Prefecture, Yunnan China; 3 Division of Chronic Infectious Disease Prevention and Control, Xishuangbanna Prefectural Center for Disease Control and Prevention, Jinghong, Xishuangbanna Dai Autonomous Prefecture, Yunnan China

## Abstract

The influence of meteorological determinants on tuberculosis (TB) incidence remains severely under-discussed, especially through the perspective of time series analysis. In the current study, we used a distributed lag nonlinear model (DLNM) to analyze a 10-year series of consecutive surveillance data. We found that, after effectively controlling for autocorrelation, the changes in meteorological factors related to temperature, humidity, wind and sunshine were significantly associated with subsequent fluctuations in TB incidence: average temperature was inversely associated with TB incidence at a lag period of 2 months; total precipitation and minimum relative humidity were also inversely associated with TB incidence at lag periods of 3 and 4 months, respectively; average wind velocity and total sunshine hours exhibited an instant rather than lagged influence on TB incidence. Our study results suggest that preceding meteorological factors may have a noticeable effect on future TB incidence; informed prevention and preparedness measures for TB can therefore be constructed on the basis of meteorological variations.

## Introduction

Tuberculosis (TB) is a widely distributed infectious disease, caused by *Mycobacterium tuberculosis*; roughly one-quarter of the world’s population has been infected^1^. Among all infectious diseases, TB is the second most common cause of death, after HIV/AIDS^2^. As a global public health challenge in general, geographical disparities are also prominent in the TB burden, with most newly diagnosed cases and deaths occurring in developing countries in Africa and Asia, such as India, Indonesia, Nigeria, Pakistan and China^3^. Although huge progress has been achieved in reduction of TB-associated mortality, currently China still has the third largest number of TB cases globally, accounting for around one-tenth of the world’s total^3^.

It has long been suggested by epidemiological studies that meteorological factors, typically temperature, humidity and wind, can influence the incidence of infectious diseases. Considering the continuous variation of meteorological factors in the time dimension, their possible influences on infectious disease incidence have been estimated recently by using a time series approach, rather than merely calculating the marginal association from traditional univariate or multivariate statistical models. For example, Huang *et al*. applied a generalized additive model (GAM) to investigate the effect of meteorological factors on the incidence of hand, foot, and mouth disease (HFMD) in Chinese children^4^, Goto *et al*. used a linear regression model in combination with a vector autoregressive (VAR) model to evaluate the influence of weekly average maximum temperature and total rainfall on Dengue fever incidence^5^, and Duan *et al*. adopted an expanded autoregressive integrated moving average (ARIMA) model to study the impact of meteorological changes on scarlet fever incidence^6^.

Although spatial–temporal characteristics of TB incidence have been investigated by commonly used time series models, such as ARIMA, seasonal autoregressive integrated moving average (SARIMA), and nonlinear autoregressive neural networks^7,8^, the influence of meteorological determinants remains severely under-discussed. Previously published studies suggested that, in many countries, including China, the incidence of TB presented obvious periodic and seasonal features, which indicates that the occurrence of TB might be significantly influenced by meteorological determinants^9,10^. Therefore, understanding the spatial-temporal association between meteorological factors and TB incidence can provide important information for targeting intervention measures of TB. However, after thorough literature review, we identified only two pertinent studies: in one study, following application of a spatial panel data model, the authors reported that temperature, precipitation, and wind speed were significantly associated with TB incidence; in the other study, an unmeasured component model (UCM) revealed an identifiable cross correlation between historical sunshine data and TB incidence^11,12^. No available study has ever simultaneously discussed the influence of expanded meteorological factors on TB incidence through the perspective of time series analysis, which can effectively capture and control for the autoregressive behavior of study variables in the time dimension.

In this study, in order to address the aforementioned deficiencies in the field, we aimed to explore the secular influence of multiple meteorological indicators on the incidence of TB in an ethnic locality in Southwest China, by using consecutive surveillance data collected over 10 years.

## Materials and Methods

### Study site

**Figure 1:**
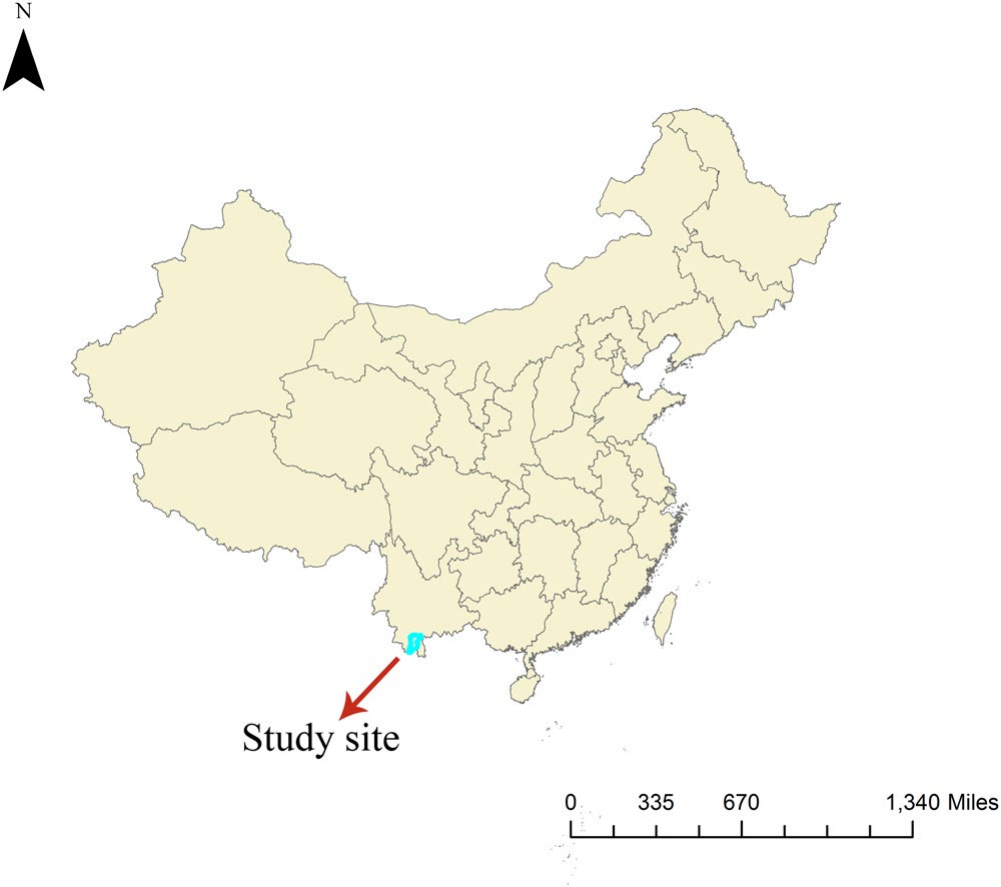
Geographical location of study site (Created by ArcMap 10.2).

We chose Jinghong city, which is located in Yunnan province in Southwest China, as our study site. Figure 1 shows the geographical location of Jinghong city. It is the capital city of Xishuangbanna Dai (an ethnic minority in China) Autonomous Prefecture, and lies in the southern corner of Yunnan province. It borders Myanmar to the south, and has an area of 6,958 square kilometers and a population of about 0.52 million. Jinghong has a northern tropical and southern subtropical humid monsoon climate: the yearly average temperature ranges between 18.6 °C and 21.9 °C, annual precipitation is 1,200 to 1,700 millimeters, annual sunshine 1,800–2,300 hours, and annual relative humidity is 80–86%.

### Data resource

The monthly reported TB incidence rate in Jinghong city from January 2006 to December 2015 was obtained from the China Information System for Disease Control and Prevention, a national online instant reporting system for notifiable infectious diseases. Monthly meteorological information for the same period was provided by the Jinghong Municipal Meteorology Bureau. Eight indicators were chosen: minimum temperature, maximum temperature, average temperature, total precipitation, maximum precipitation, minimum relative humidity, average wind velocity, and total sunshine hours.

### Statistical analysis

Rather than using raw data, it was thought to be much more meaningful to investigate the influence of meteorological variation on fluctuations in TB incidence, thus we differenced the original time series of TB incidence and eight meteorological factors, and constructed nine secondary time series for further analysis. The stationarity of each secondary time series was tested by using the augmented Dickey–Fuller test. Lagged cross correlations between the stationarized TB incidence series and each of the meteorological factor series were calculated for lags up to 4 months, in consideration of monthly-based and differenced nature of the analyzed time series, more lags will bring about the concern of sparse data and influence statistical efficiency in estimation. A previously published study suggested that, when estimating cross correlations between two time series, autocorrelation should be controlled for simultaneously. Otherwise, spurious cross correlation is likely to be found^13^. Thus we used the autocorrelation function (ACF) to evaluate the autoregressive nature of the differenced TB series.

Subsequently, in consideration of the lagged and usually nonlinear relationship between weather factors and health outcomes, we applied a distributed lag nonlinear model (DLNM) to explore the influence of the chosen meteorological indicators on TB incidence. The DLNM is a novel and flexible modeling framework for dealing with lagged nonlinear associations between or among time series sequences. When using DLNM, a conceptual two-dimensional space is constructed initially, the two bases of this space corresponding to the predictor and lags, respectively. After that, the associations between the variable of interest and these two bases are estimated simultaneously, usually by using nonlinear algorithms such as polynomials, natural cubic spline, B-spline, etc. A thorough methodological description and R code of this method was given by Gasparrini^14^. In this study, we used the 3^rd^ order polynomial for both the lagged and nonlinear influences of the chosen meteorological factors. This algorithm was chosen because of its flexibility and parsimony, as suggested by a previous study^15^. As mentioned earlier, the maximum lag for all predicting factors was set as 4 months. Since a preliminary analysis revealed expanded correlations between most of the meteorological indicators, including them simultaneously in the same model would have caused the problem of collinearity, thus we fitted a series of DLNMs separately for each of the indicators.

The final DLNM we used can be written as:

1 $${\rm{Diff}}{({\rm{Incidence}})}_{{\rm{t}}}={{\rm{r}}}_{0}\sum _{{\rm{l}}=1}^{{{\rm{l}}}_{{\rm{\max }}}}{\rm{f}}({\rm{Diff}}{({\rm{x}})}_{{\rm{t}}-1},{{\rm{r}}}_{{\rm{t}}-1})+\sum _{{\rm{i}}=1}^{{\rm{n}}}{{\rm{r}}}_{{\rm{i}}}{({\rm{Incidence}})}_{{\rm{t}}-1}$$

In model (1), Diff (Incidence)_t_ is the difference in TB incidence at time t, r_0_ is the intercept of the model, Diff(x) is the difference in a certain meteorological indicator, and f (Diff(x)_t−1_, r_t−1_) describes the specific nonlinear function between indicator and dependent variable at a certain time lag *t* − *l*. l_max_ denotes the maximum lag period $\,\sum _{{\rm{i}}=1}^{{\rm{n}}}{{\rm{r}}}_{{\rm{i}}}{({\rm{Incidence}})}_{{\rm{t}}-1}$ represents the collective effect of identified autocorrelation series, n denotes the number of auto-correlated independents. Given that we intended to fit the DLNM on the basis of a differenced stationary time series, it was not necessary to incorporate secular trend and seasonal variation in the final model.

All statistical analysis was conducted using R software (Version 3.3.3, The R Foundation for Statistical Computing, Vienna, Austria). The significance level for each statistical test or inference was set as a two-tailed probability of less than 0.05.

### Ethics statement

This study was approved by the Ethics Committee of Kunming Medical University. All methods were performed in accordance with the relevant guidelines and regulations. Informed consent for study participation was not required because all analysis was based on aggregate data which did not contain individually sensitive information.

### Data availability

The datasets analyzed during the current study are not publicly available owing to a confidentiality agreement with collaborative institutions, but are available from the corresponding author on reasonable request.

## Results

### Initial sequences of TB incidence and meteorological factors

**Figure 2:**
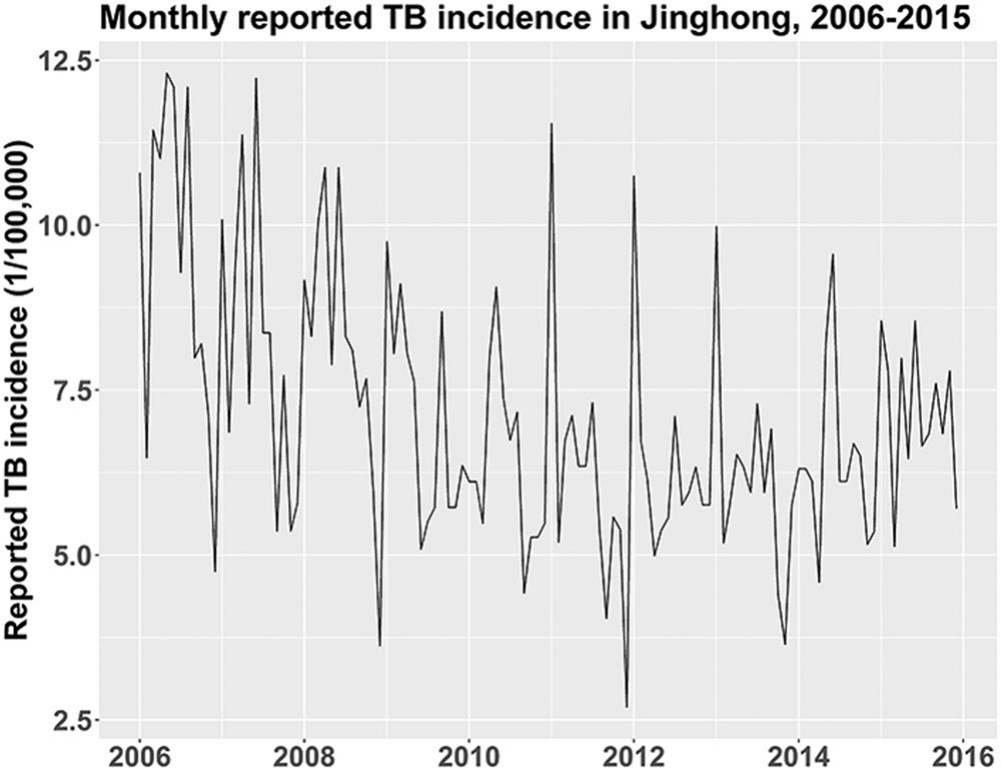
Monthly reported TB incidence in Jinghong, China, 2006–2015.

**Figure 3:**
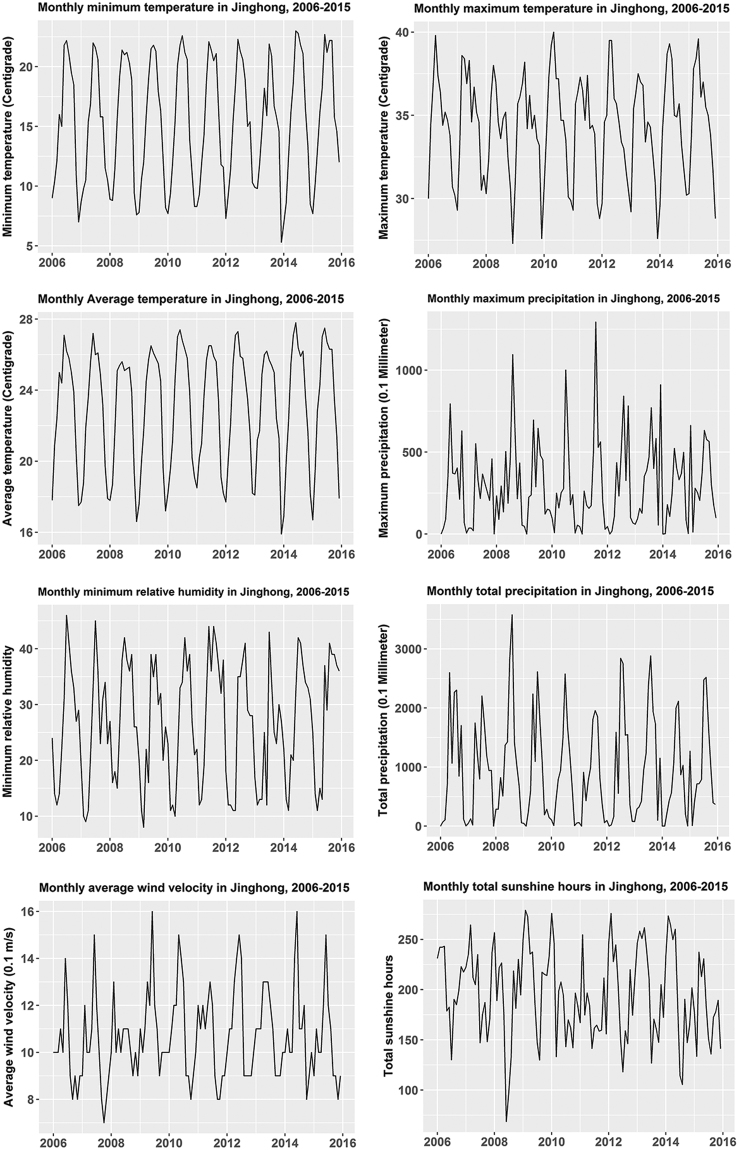
Monthly data on meteorological factors in Jinghong, China, 2006–2015.

The monthly TB incidence fluctuated between 2.69 and 12.31 per 100,000 population in Jinghong from 2006 to 2015, with a median of 6.70 per 100,000. The minimum and maximum annual TB incidences were 73.66 and 113.60 per 100,000, respectively, with a median of 81.23 per 100,000. The annual TB incidence generally presented a decreasing trend; nevertheless, a noticeable rebound was discerned from 2013 to 2015 (Fig. 2). Variations of the eight meteorological factors with time were compiled in Fig. 3: all indicators basically presented a recognizable annual cyclic pattern, especially the minimum temperature, maximum temperature, average temperature and minimum relative humidity.

### Cross correlations between TB incidence and meteorological factors

The results of the augmented Dickey–Fuller test revealed that, after taking the first difference as mentioned previously, the TB incidence series and the series of all meteorological factors were stationary for further analysis (Table 1). Lagged cross correlation coefficients were calculated and are listed in Table 2. Within the chosen lag period of 4 months, among all eight meteorological factors, only the changes in two humidity related indicators at lag 4, total precipitation and maximum precipitation, were significantly cross correlated with TB incidence variation.

**Table 1:** Augmented Dickey–Fuller test results for differenced time series.

Differenced time series	Statistic	*p* value	Conclusion
TB incidence	−19.58	<0.01	Stationary
Minimum temperature	−7.16	<0.01	Stationary
Maximum temperature	−9.32	<0.01	Stationary
Average temperature	−5.69	<0.01	Stationary
Total precipitation	−13.20	<0.01	Stationary
Maximum precipitation	−17.12	<0.01	Stationary
Minimum relative humidity	−11.65	<0.01	Stationary
Average wind velocity	−11.88	<0.01	Stationary
Total sunshine hours	−13.35	<0.01	Stationary

**Table 2:** Lagged cross correlation coefficients between TB incidence and meteorological factors.

Lags	MINT	MAXT	AVET	TP	MP	MRH	AWV	TSH
Lag 0	0.05	−0.02	0.02	0.07	0.12	−0.06	0.18	0.09
Lag 1	−0.07	0.08	−0.03	−0.04	−0.09	0.05	−0.01	−0.07
Lag 2	−0.02	−0.09	−0.11	−0.11	−0.11	−0.14	0.07	0.06
Lag 3	−0.18	0.03	−0.03	0.07	0.18	−0.01	−0.15	−0.02
Lag 4	−0.02	0.05	−0.05	−0.25*	−0.36*	−0.17	0.11	0.10

MINT: minimum temperature; MAXT: maximum temperature; AVET: average temperature; TP: total precipitation; MP: maximum precipitation; MRH: minimum relative humidity; AWV: average wind velocity; TSH: total sunshine hours.

*p < 0.05.

### Lagged nonlinear associations between meteorological factors and TB incidence

**Figure 4:**
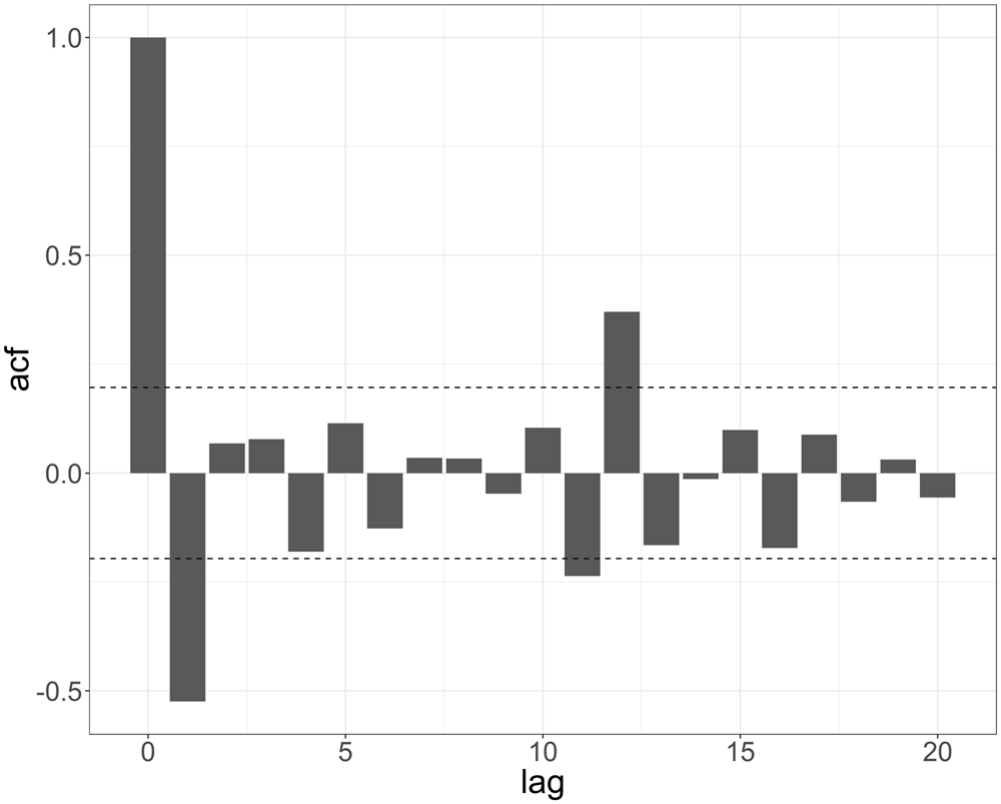
Autocorrelation function (ACF) graph of differenced TB incidence sequence.

**Figure 5:**
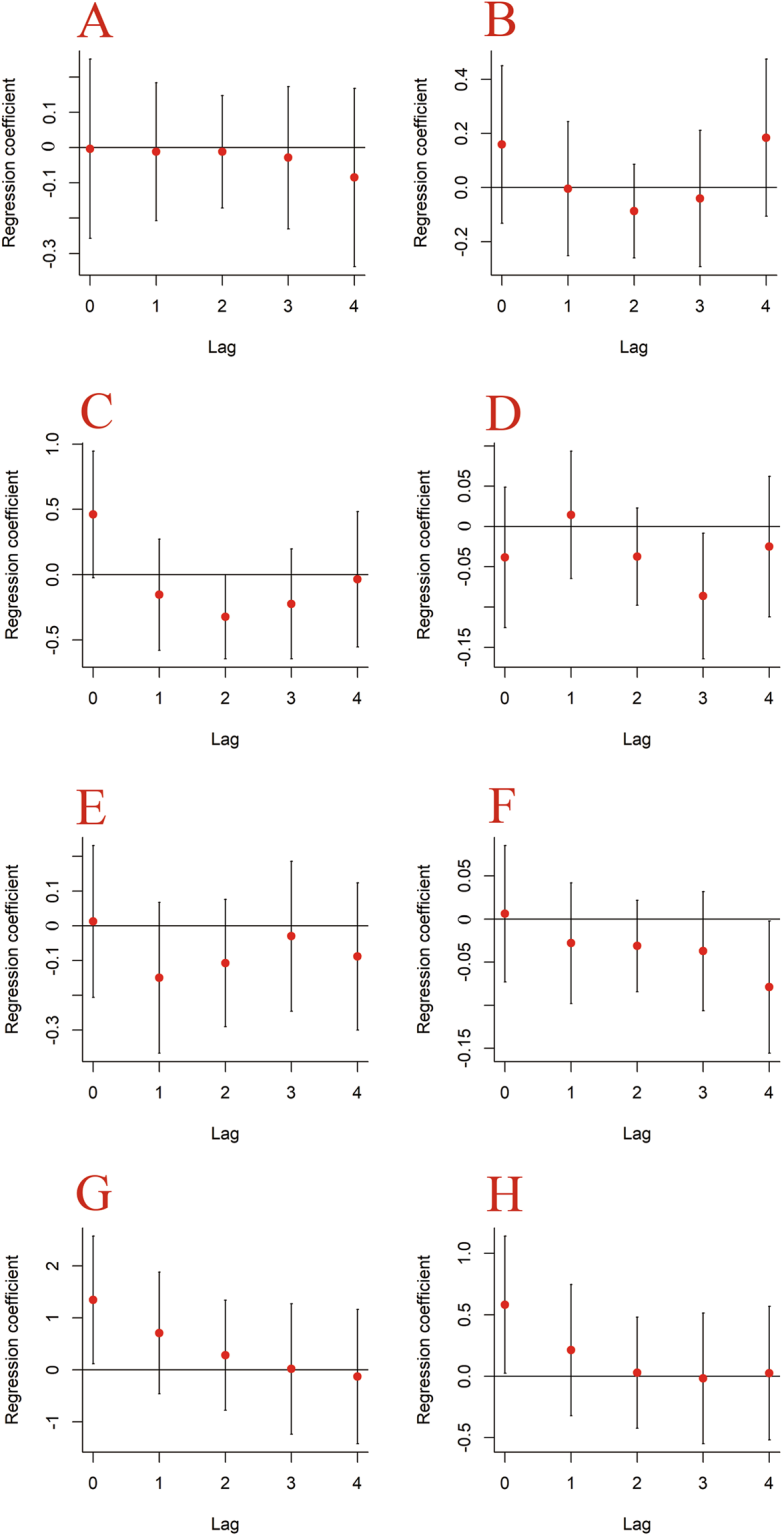
Distributed lagged nonlinear associations between meteorological factors and TB incidence. A represents minimum temperature (every 1 °C increase), B represents maximum temperature (every 1 °C increase), C represents average temperature (every 1 °C increase), D represents total precipitation (every 1 °C increase), E represents maximum precipitation (every 1 centimeter increase), F represents minimum relative humidity (every 1 percent increase), G represents average wind velocity (every 1 meter/second increase), H represents total sunshine hours (every 40 hours increase). The error bounds reflect 95% CIs.

The ACF graph revealed that TB incidence at the previous lag was significantly associated with current TB incidence, with an autocorrelation coefficient of −0.524 (*p* < 0.05) (Fig. 4). DLNMs that incorporated the identified autoregressive pattern of the TB incidence series were built separately for each meteorological indicator, and the model fitting results are summarized in Fig. 5. When compared with the results shown in Table 2, and after controlling for autocorrelation, five meteorological indicators were significantly associated with TB incidence variation at different time lags: average temperature at lag 2, total precipitation at lag 3, minimum relative humidity at lag 4, and average wind velocity and total sunshine hours at lag 0. With regard to the patterns of correlation, average temperature, total precipitation, and minimum relative humidity were negatively correlated with TB incidence, whereas average wind velocity and total sunshine hours were positively correlated with TB incidence.

## Discussion

In this study, through the perspective of time series analysis with the use of DLNM, we explored the influence of multiple meteorological factors on TB incidence by using a 10-year set of consecutive surveillance data. Based on the analytical results, we found that, in general, the fluctuations of meteorological factors related to temperature, humidity, wind, and sunshine were associated with TB incidence variation. Nevertheless, the patterns and magnitude of the associations were disparate.

Among all the temperature indicators, only the average temperature 2 months ahead was significantly associated with TB incidence variation in the current month. Moreover, the cross correlation between TB incidence and the average temperature was inverse, based on the analytical results, which suggests that an increase in the average temperature could bring about a decrease of TB incidence in the future. A previously published study also analyzed the association between monthly average temperature and TB incidence and reported similar findings^16^. Nevertheless, that study estimated the association on the basis of the absolute values of TB incidence and temperature, and the statistical models the author applied did not effectively correct for possible autocorrelation. In comparison, the difference-based DLNM model we adopted is more appropriate for determining the influence of temperature, and the explanation of the results seems more intuitive. Although several suggestions can be made to explain this lagged influence of temperature on TB incidence, the most plausible is that temperature directly changes the timing of indoor/outdoor activities of both TB susceptible and infected populations, and thus influences the transmission probability of *Mycobacterium tuberculosis*^17,18^. This may also explain why the other temperature indicators we investigated, such as minimum and maximum temperature, were not significantly associated with TB incidence. It is intuitive that the level of outdoor activities will be influenced predominantly by the average temperature condition in a given period. The evidence that the median time interval between TB infection and an observable immune response is commonly about 7 weeks indirectly supports this 2-month lagged influence^19^. However, the rationale behind this inverse association should be further investigated and explained because, unlike many other airborne infectious diseases, the peak of TB incidence in many countries usually occurs during summer, with high temperatures, instead of in winter or spring^20,21^. Moreover, although some may quickly develop into active patients after TB infection, a part of infected population will experience considerably long latent periods up to years or even decades^22^. Therefore, it is possible that this comparatively short-term association may reflect the influence of the average temperature on the activation of TB among those latent patients.

Another major finding of this study is that humidity related indicators such as total precipitation and minimum relative humidity presented a long-term lagged effect on TB incidence. The correlations between humidity related factors and TB incidence we found were all inverse, which indicates that a drier climate was likely to be followed by a higher incidence of TB. The association between humidity and TB incidence was also discussed by two newly published articles, albeit different statistical methods were applied. Cao *et al*. examined this association by using linear regression Bayesian models and reported that average humidity was inversely associated with TB incidence in mainland China; however, this inverse association was not statistically significant^23^. Beiranvand *et al*. concluded that the average annual rainfall, another direct indicator of humidity, inversely influenced the TB incidence rate in an Iranian province between 2005 and 2012, on the basis of linear regression analysis^24^. Our study results were comparable to these two studies. A possible explanation is that long-term exposure to dry air may reduce the production of protective mucus on the surface of the respiratory tract, thus reducing its resistance to the growth of *Mycobacterium tuberculosis*, as suggested by laboratory findings^25^. Other plausible mechanisms may also exist and remain to be discovered. Also, the other explanation that humidity triggered the activation of latent TB could be possible.

One interesting finding of our study was that two meteorological indicators, wind and sunshine, exhibited an instant rather than lagged association with TB incidence. Both influenced TB incidence positively, which means that, with increased wind speed or intensification of solar radiation, TB incidence was simultaneously increasing. Considering that any influence on a communicable disease such as TB will inevitably occur by affecting three major segments (source of infection, route of transmission, and susceptible population), it will generally take some time for the disease to incubate and signs to develop. Therefore, with regard to meteorological factors, it is more logical to expect a lagged correlation with TB incidence. We suspect that the instant correlations we found probably represented a kind of “accompanying association”. In a 2013 study, Koh *et al*. found that the decrease in total sunshine hours in winter was correlated with peaks in TB incidence 6 months later^12^. The increase in solar radiation is also usually seen in the summer in Jinghong, therefore the simultaneously increasing trend of TB incidence may actually be caused by deficient sunshine in the previous winter. One plausible mechanism is that reduced sunshine exposure can lead to decreased vitamin D levels, which impairs host immunity to infectious agents: a previously published systematic review summarized a strong link between vitamin D deficiency and TB prevalence^26^. Similarly, the windy season in Jinghong usually starts in February and peaks in June. The accompanying increase in TB incidence in this period may also be a product of dropping temperatures traced back to the winter season. Nevertheless, this “accompanying association” and postulations on its causes should be corroborated by future studies with more extended data in the time dimension.

Several limitations of this study should be noticed. First, our DLNMs were based on monthly time series sequences. Although monthly data are widely used in discussing cross correlations between time series sequences, measurements based on such long time intervals may be less accurate, and therefore the risk of biased or even misleading results cannot be precluded. Future time series studies on a weekly or even daily basis are required to corroborate our major findings. Second, when estimating the associations between the chosen meteorological factors and TB incidence, because of the likely collinearity suggested by the data we adopted univariate rather than multivariate statistical models; therefore, it is possible that for some indicators the results were distorted by uncontrolled residual confounding. Finally, given the ecological nature of the study, although the time sequence is clear, causal inference is inappropriate.

To conclude, our study investigated the influence of multiple meteorological factors on TB incidence by using 10-year surveillance data in a localized region in Southwest China. Through the perspective of time series analysis, we found that temperature, humidity, wind and sunshine may influence future fluctuations in TB incidence. Our study results provide useful information to public health officials in formulating targeted prevention and preparedness measures.
